# Stimulating Contributions to Public Goods through Information Feedback: Some Experimental Results

**DOI:** 10.1371/journal.pone.0159537

**Published:** 2016-07-26

**Authors:** Marco A. Janssen, Allen Lee, Hari Sundaram

**Affiliations:** 1 School of Sustainability, Arizona State University, Tempe, Arizona, United States of America; 2 Center for Behavior, Institutions and the Environment, Arizona State University, Tempe, Arizona, United States of America; 3 Department of Computer Science, University of Illinois at Urbana-Champaign, Urbana, United States of America; Institutes for Behavior Resources and Johns Hopkins University School of Medicine, UNITED STATES

## Abstract

In traditional public good experiments participants receive an endowment from the experimenter that can be invested in a public good or kept in a private account. In this paper we present an experimental environment where participants can invest time during five days to contribute to a public good. Participants can make contributions to a linear public good by logging into a web application and performing virtual actions. We compared four treatments, with different group sizes and information of (relative) performance of other groups. We find that information feedback about performance of other groups has a small positive effect if we control for various attributes of the groups. Moreover, we find a significant effect of the contributions of others in the group in the previous day on the number of points earned in the current day. Our results confirm that people participate more when participants in their group participate more, and are influenced by information about the relative performance of other groups.

## Introduction

There is a substantial understanding of the conditions that lead to successful governance of the commons by small groups such as communities [1]. Studies in small-scale communities and in controlled experiments [2] show that the strength of groups in overcoming collective action problems lies in whether or not participants can communicate, whether they have input in the creation of the rules, whether there is group homogeneity, and whether institutional arrangements are monitored and enforced. In small-scale communities, participants have relatively low costs in deriving information to determine the trustworthiness of others. Small communities are also characterized by low participant costs for monitoring others’ behavior, as well as low costs for face-to-face meetings. The low costs of monitoring behavior and conducting face-to-face meetings are not generally possible at a large scale. Identifying how to scale up the insights that lead to success at the community level to larger scale collective action problems remains a fundamental challenge. Addressing a global scale problem like climate change requires actions at different levels of scales [3], including bottom-up initiatives in a polycentric system [4].

Computational tools have the potential to play an important role in addressing larger-scale challenges by mitigating transaction costs, developing more efficient trust mechanisms, and addressing heterogeneity. Computational infrastructures like social networking sites reduce communication costs by enabling individuals at different geographical locations to message one another with minimal cost. Low-cost physical-world sensors such as smart meters enable individuals to understand energy use in real-time, as well as the activity breakdown [5,6]. The low communication costs and the low sensing costs allow for rapid delivery of information, thus providing crucial real-time feedback on the consequences of our decisions and the decisions of other participants. Examples include smart energy meters (e.g., [7]), smart water meters (e.g., [8]), tracking locations (e.g., [9]), and remote sensing of heat loss (e.g., [10]).

Second, with increased participation in online networks, new forms of “trust” begin to emerge, and there are computational mechanisms to extract smaller homogenous communities from large groups. In social networks, such as Twitter (http://twitter.com) and Facebook (http://facebook.com), people can passively, and asynchronously “follow” each other, creating a new form of “ambient intimacy” [11] that is unavailable with inter-personal communication in the physical world, which are typically synchronous.

Clearly, computational tools provide opportunities to scale up the strengths of self-governance observed in smaller communities. There has been significant interest in developing web and mobile applications for reducing an individual’s carbon footprint (stepgreen; http://www.stepgreen.org/), energy use (OPOWER; http://opower.com/, tendril energize; http://www.tendrilinc.com/, peoplepower; http://www.peoplepowerco.com/, joulebug; http://joulebug.com/), transport (ubigreen; https://www.cs.washington.edu/node/3862), competitive sustainability challenges (ecochallenge; http://eco-challenge.eu/en/), water use (999 bottles; https://www.artefactgroup.com/content/work/999bottles/) or sustainable behavior in general (green; http://www.practicallygreen.com/, eEcosphere; http://www.eecosphere.com/, Rippl; http://www.oceanconservancy.org/do-your-part/rippl.html) (see also [12,13]).

There has been some analysis of the effectiveness of these recent technologies. [14] evaluated the effectiveness of a sample of users via self-reports. A more systematic analysis has been performed with OPOWER for a few hundred thousand households [15]. A significant reduction of energy use of around 2% has been found due to providing social feedback on energy bills. One of the challenges in measuring the effect of these apps and websites is the issue of verification of a user’s actual behavior. [16] provide another example where information about voting behavior of friends on Facebook led to a significant increase of 0.37%. Disaggregated energy use is possible with new smart meters [5,6] but it is much more difficult to monitor the many other behaviors (for example, eating vegetables instead of beef) affecting our resource use.

These new developments led us to design a new type of experiment to test the effectiveness of different incentives on participant behaviors over a number of days. We developed a web-based experiment environment capable of running public good experiments over several days with large groups of participants.

Traditional public good experiments typically have groups of two to ten participants who come to a designated laboratory and make decisions on how much of an experimenter-provided endowment should be invested in the public good, and how much to keep for themselves [17]. The total investment in the public good is then multiplied and shared equally among the participants. The best outcome for the group would be for every participant to invest the whole endowment. The best material outcome for an individual would be to free ride on the actions of the others and not invest in the public good. We typically see an initial level of contributions around 50%, which then declines over subsequent rounds [18]. In recent work, researchers have run web-based experiments using Amazon’s Mechanical Turk [19–21]. In these experiments participants must all log in at the same time to participate for an hour, similar to traditional one-hour experiments run in economics labs.

We are developing a mobile app to perform experiments on the role of social information on physical actions to improve sustainability outcomes. With this mobile app physical actions can be verified via photos or location information. At this moment a reliable implementation of verification of physical actions is not finished, and therefore we will have participants performing virtual actions to test initial hypotheses. Hence the reward for the participants depends only on their own efforts and the efforts of their group members to login to the web-based environment at the right time. We used student subjects at our university campus for logistical reasons as we aim to continue those experiments with verification of physical actions. For such verification we would initially work at our university campuses. For example, we will partner with local initiatives that could facilitate the verification process (scanning a barcode when a participant returns batteries). We recognize that our design has limitations by using student participants and virtual actions. Therefore the results will not be representative for the general population. But a study like this is needed in the pathway towards a more comprehensive design.

Although we are developing specialized experiment infrastructure, we also recognize the value of existing websites that promote large-scale behavioral change. We are also studying collective action in online communities to complement our controlled experiments [22]. This online community research has shown that there are many challenges in capturing participant attention and retention and that successful communities often have a portfolio of tasks to entice active and sustained participation.

In this paper, we report on the first of a series of experiments with our infrastructure. In traditional one-hour experiments participants receive an endowment that they can invest in the public good or keep for themselves. In our experiment, participants do not receive an initial endowment. Instead, they must invest a small amount of their time repeatedly throughout a period of five days to contribute to the public good by logging into the website on their laptop or smart phone, click on the virtual activities that are available to them at that time. In this sense, a participant’s time is their natural endowment and participation in the experiment competes with the participant’s other activities they are involved in. Since participants have to take time each day to make a contribution to the public good they have to remain motivated to participate.

Our experimental goal for this paper is to replicate a finding from a traditional public good experiment. [23] found that if groups receive information that compares their performance to other groups—where the information does not affect their material rewards—the comparative information leads to an initial increase in contributions. Over the long term, the benefit from comparative information disappears. In our experiment we will test the effects of information feedback on the level of participation.

We will now discuss the experimental design, before we discuss the results. The paper will close with a discussion of the possibilities of web-based experiments to test collective action for groups and social networks.

## Experimental Design

The experiment is based on a linear public good problem [17]. The theoretical formulation is as follows: The monetary reward is linear to the contributions of all N individuals of the group. As mentioned earlier, participants do not receive a monetary endowment, but invest their time. Participants may invest an amount equal to *x* minutes to the public good, and may receive *y* dollars dependent on the points collected on average by all group members. The value of time might not be the same for every participant. Hence there will be a natural heterogeneity among the participants in evaluating the time commitment with respect to the expected rewards. Note that in traditional experiments there is also heterogeneity with respect to how participants value a dollar from the experiment. Since the experiment is run over several days, we expect that participation over time will be adjusted based on the expected rewards.

**Fig 1:**
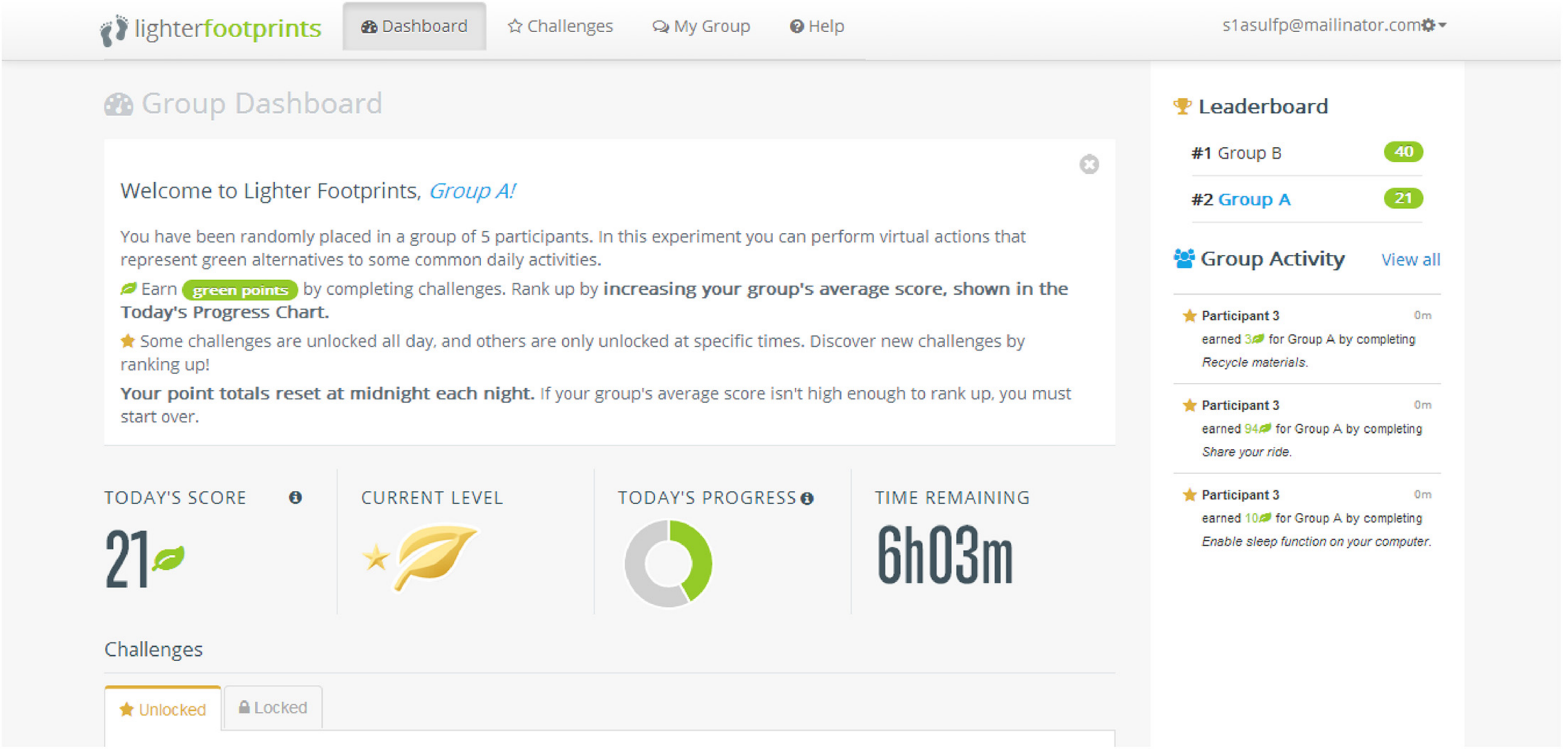
Screenshot of the Lighter Footprints experiment. Participants can view their group progress and the leaderboard comparing their group performance with other groups.

**Fig 2:**
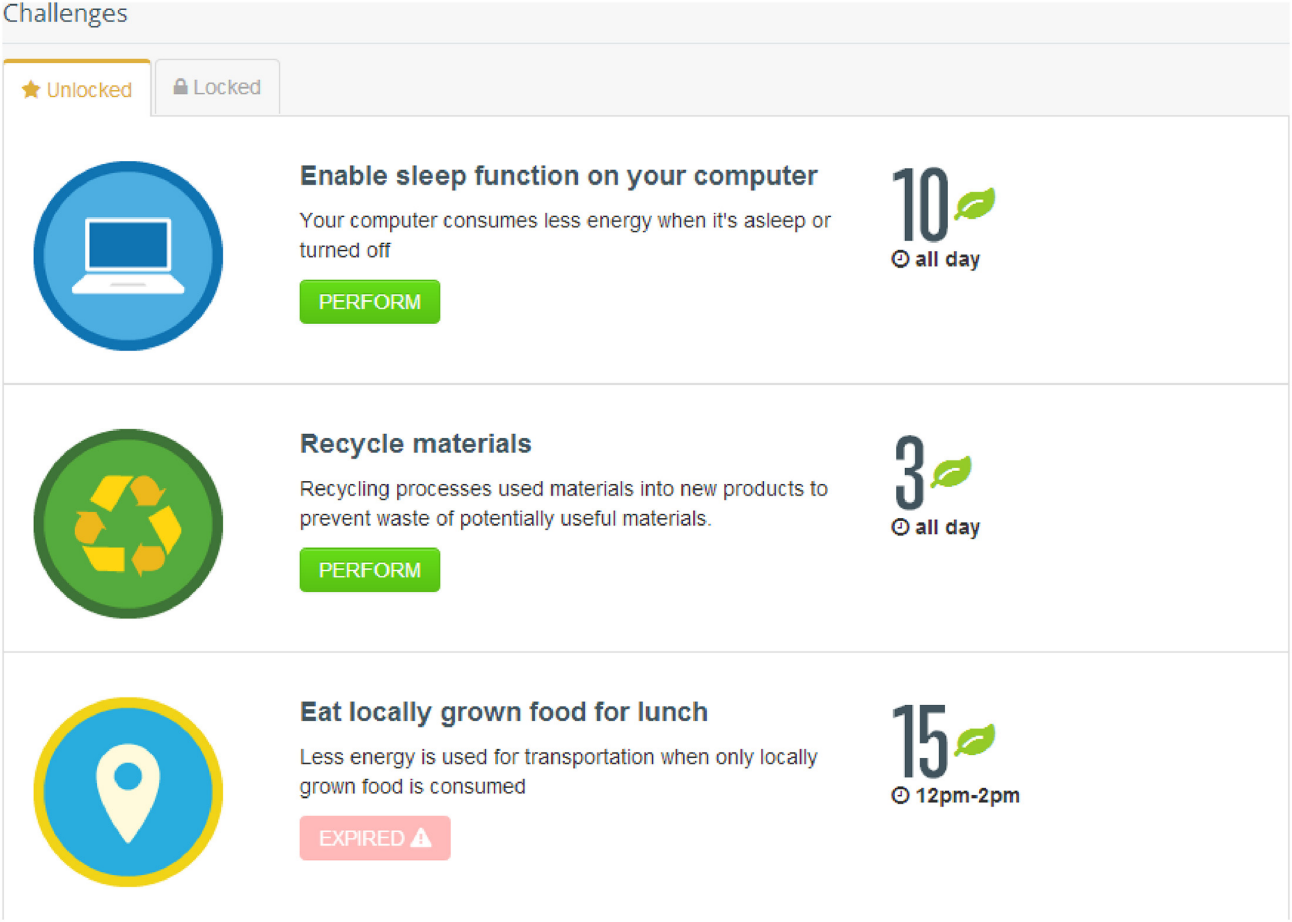
Screenshot of some of the actions the participants can take. If an action is still available the button is green and the action can be performed by clicking on the button. If the action is not available anymore, it is showed by a red button with the text “expired”.

Our experiment is framed as a carbon footprint reduction game where participants can perform virtual actions representing sustainable alternatives to common activities during a 5-day period. These sustainable alternatives are only available at certain time intervals throughout the day, coinciding loosely with when those activities are available (e.g., carpooling is available between 8–10 AM and 4–6 PM, local Phoenix time). Participants login to a website where they can view an update of their group’s progress (Fig 1) and the activities that are currently available (Fig 2). Each activity performed–by clicking on a button on the website—generates points for the group if the participant clicks on the button when the activity is available. This version of the experiment only requires participants to click on the actions. They do not have to actually perform those actions in real life. In essence, this public good game tests whether enough participants within a group will log in to the website at the right time to click on the Perform button for an available activity, contributing their time to the public good.

The reason we frame this public good experiment as a carbon footprint reduction game is to make it less abstract and more compelling for participants. We also found in tests with earlier versions of the experimental environment that the website design needed to be engaging and clear in order to maintain participant interest. This also meant that we allow participants to leave chat messages to other group members, and to like the actions of other members of the group. This may allow for group coordination, and team building.

Participants in laboratory experiments are making decisions for a limited amount of time and are monitored during the duration of the experiment. Hence in laboratory experiments we expect participants to pay attention to the experiment even if the task is abstract. In our web-based experiment, the participation in the experiment competes with other tasks students may engage in. Hence we had to make the experiment less abstract. The final design was the result of involvement of undergraduate student web developers and feedback from pretests in two large undergraduate courses. When Amazon Turk is used the commitment is somewhat lower than laboratory experiments, but 90% of the participants still remain in the experiment until the end [24]. In our design, the experiment naturally competes with the various other activities a participant has going on in their daily life. Since participants still receive daily emails, we consider all participants in the experiment for the duration of five days. The experiment was implemented using the vcweb framework (http://vcweb.asu.edu/).

At the start of the experiment, registered participants are sent an email with their username and password to join the experiment. Once logged in they can view the currently available activities (Table 1), select an activity to perform and earn points for their group. Earnings are based on the accumulated points per person over the week. The rewards are 2 cents per point which leads to maximum earnings of 25 dollars. The amount of accumulated money earned is shown directly on the participant’s home dashboard.

**Table 1:** Activities for the different levels.

Activity	Points	Time Activity is Available	Day 1	Day 2	Day 3	Day 4	Day 5
Enable sleep function on computer	10	All day	X	X		X	
Eat local food lunch	15	Noon– 2pm	X		X		
Carpool	94	8am– 10am and 4pm– 6pm	X	X	X	X	X
Adjust thermostat by 2 degrees	55	6am-8am		X	X	X	X
Recycle materials	3	All day		X	X		
Turn off the water when you brush your teeth	8	7am-9am and 10pm-12pm		X			
Bike or take public transport to go out	75	6pm-11pm	X	X	X	X	X
Recycle newspaper	6	All day	X				
Turn off computer during the night	14	Midnight-8am		X			X
Replace beef with poultry	43	6pm– 7pm				X	
Turn off the lights if you leave a room	23	6pm– 11pm		X	X		
Green lunch	14	Noon-2pm	X				
Wash with cold water	2	4pm-11pm	X		X	X	
Air dry your clothes	20	Midnight-6am	X				
Vegan breakfast	44	7am-9am			X		X

Points for each activity are earned when a participant logs into the web application and presses the “Perform” button during the time slot when the given activity is available. One point roughly corresponds to 0.1 pound of CO_2_ a day saved. Information on the relation between points and pounds of CO_2_ saved is available to the participants in the experiment.

**Fig 3:**
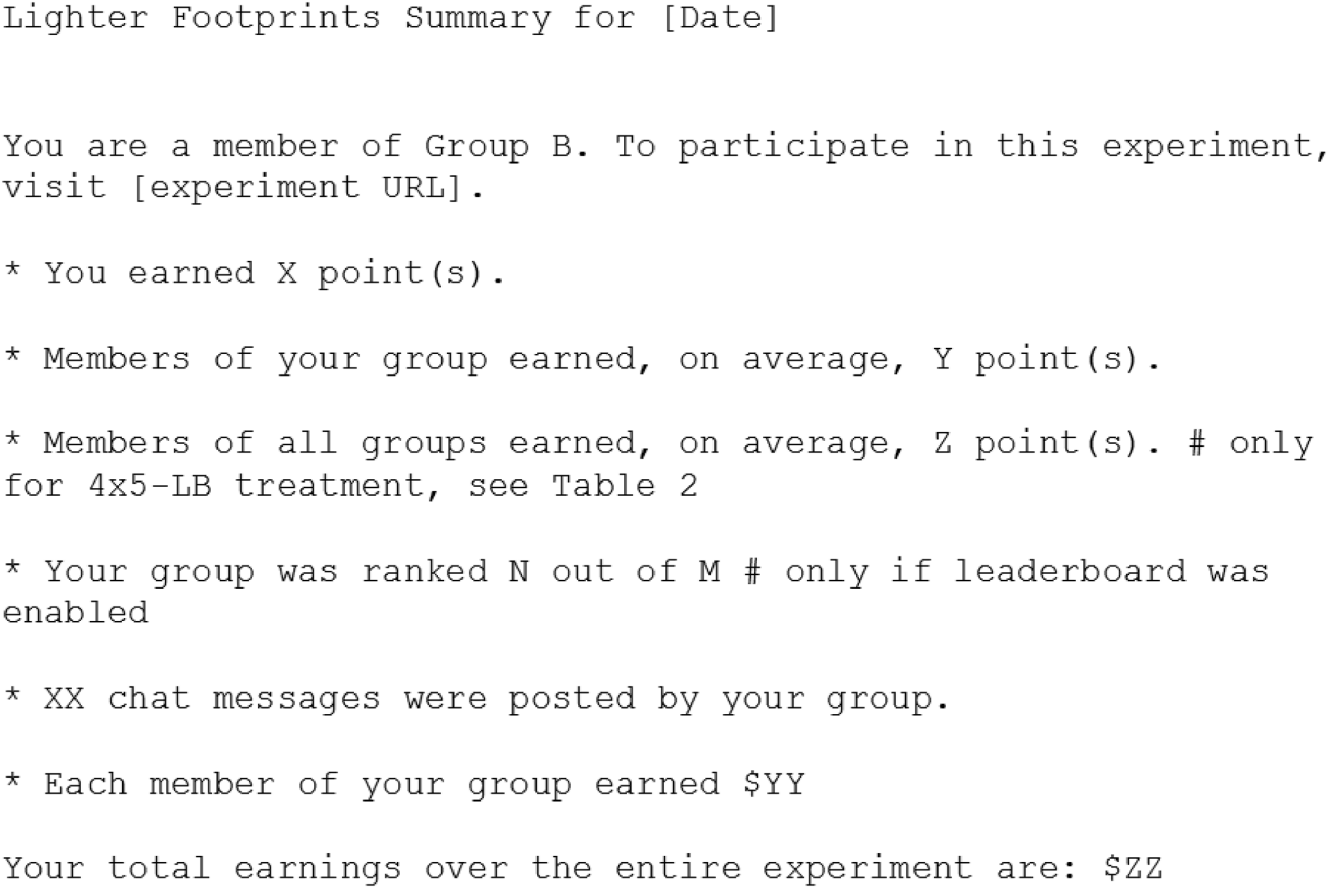
Text of the nightly email.

We developed four treatments based on [23]. [23] showed that providing information about group performance compared to other groups temporarily increases the level of cooperation. Group comparison was implemented by adding a leaderboard on the front page of the experiment website (see Fig 1). We also sent a nightly email to each participant that summarized their group’s results for the day. The text of the nightly email is shown in Fig 3.

We considered four treatments (Table 2). The reason for those four treatments is to test the effect of group size, and the effect of including leaderboard to see group performance relative to other groups. We will test leaderboards when group earnings are independent of each other, and if earnings of the groups are dependent on each other. The basic two treatments are groups of 5 with and without a leader board (5-LB and 5-NLB). In 5-LB there are 20 groups of 5 in the experiment at the same time. Hence the participants can see how their group is performing compared to 19 other groups. In the treatment 5-NLB there are also 20 groups in the experiment at the same time, but they do not receive information about the performance of the 19 other groups. Those two treatments allow us to test the effect of leaderboards for small groups, similar to [23]. We performed different sessions leading to 60 groups in treatment 5-LB and 40 groups in treatment 5-NLB.

**Table 2:** The basic information of the four treatments.

Treatment	Description	Individual level information about how many persons and groups	Group size from which the rewards are calculated	Number of participants	Number of groups
5-LB	5 person groups who can see their relative score (Leader Board) among 20 groups during the experiment. Earning is based only on decisions of own group of 5 individuals.	5 individuals/20 groups	5	300	60
5-NLB	5 person groups who do not derive feedback on their performance compared to others. Earning is based on decisions of group of 5 individuals.	5 individuals	5	200	40
20 –NLB	Group of 20 without leaderboard. Earning is based on decisions in group of 20 individuals.	20 individuals	20	200	10
4x5-LB	Group of 20 where 4 subgroups of 5 derive feedback how their subgroup is doing compared to other 3. Earning is based only on decisions in group of 20 individuals.	5 individuals/4 groups	20	200	10
	Total			900	120

We also wanted to test the effect of group size and performed experiments with groups of size 20 without exchanging information on the relative performance with other groups (20-NLB). Based on the classic work on collective action we would expect smaller groups would perform better compared to bigger groups [25].

Finally, we included a treatment of groups of 20 where the groups are subdivided into 4 groups of 5 (4x5-LB). The payoff depends on the performance of the group of 20, but the subgroups of 5 will see how they perform compared to the other 3 subgroups during the experiment. We call it 4x5-LB since the subgroups of 5 see their subgroup performance compared to the other 3 groups of 5. If the use of leaderboards have a positive effects this could be used to increase cooperation in public good games with larger group size. This is what we would be able to test with 4x5-LB compared to 20-NLB.

We now state the three hypotheses we test. Those hypotheses are focused on the effect of the treatments on the performance of the group over the duration of the experiment of 5 days. The hypotheses for this experiment are therefore:

H1. (5-NLB > 20-NLB) The average performance of groups of 5 is higher compared to groups of 20.
  This hypothesis is based on the seminal work of Mancur Olson [25] who argued that cooperation in public goods is higher in small groups compared to big groups.
H2. (5-LB > 5-NLB) Providing information to participants on their relative performance compared to other groups leads to higher performance of groups compared to those who do not get this information. [23] found support for H2 in their study. This hypothesis is also based on various studies that show the effect of descriptive norms (e.g. [15,16]).
H3. (4x5-LB > 20-NLB) When groups of 20 are split up in four groups with a leader board we will derive higher performance compared to group of 20 without subgroups.

Based on the arguments for H2 it would be beneficial to include group comparison. In order to reach an overarching goal for a large group one can therefore create subgroups and allow for group comparison in order to increase performance. Hence to increase the level of cooperation in a large group (20 persons in this experiment) we expect that information on the relative performance on subgroups has a positive effect.

## Results

The experimental protocol was approved by the Institutional Review Board of Arizona State University (IRB protocol # 1302008874), and the experiments were run in the Spring semesters of 2014 and 2015 and the Fall semester 2014. 900 participants were recruited from a database of potential participants for behavioral experiments among undergraduates at Arizona State University. The participants signed up the week before the experiment and were informed they would receive instructions for the web-based experiment on a Sunday evening. The participants were randomly assigned to groups and treatments. The experiment began on Monday at midnight, and ended after 5 full days passed, on Saturday at midnight. Participants were informed about the length of the experiment when they were invited to participate.

Table 3 provides the basic results of the experiments. The maximum score a group could attain in the experiment was 1250 points, and we found that all treatments averaged around 500 points. Groups of 5 without information about their relative performance had the lowest scores on average. When we use the Mann-Whitney one-tailed test on the data we find that results over the whole week are not significant from each other using a p-value of 0.05. Since 463.66 (5-NLB) is not larger than 532.27 (20-NLB) hypothesis 1 is rejected (Z = -1.52; p-value = 0.0643), meaning that we do not observe that smaller groups perform better. Although 516.21 (5-LB) > 463.66 (5-NLB) with p-value = 0.0901 (Z = -1.34), it is not statistically significant for p < 0.05 and hypothesis 2 is rejected. This means that there is no significant effect of the leaderboard. Since 524.65 (4x5-LB) > 532.27 (20-NLB) we have to reject hypothesis 3 also (p-value = 0.4247 and Z = -0.19). This means that the leaderboard has no positive effect to increase performance of large groups.

**Table 3:** Average points per person in the four treatments for the five days total and each day separate. The standard deviation is between brackets.

	5-LB	5-NLB	20-NLB	4x5-LB
Total	516.21(169.97)	463.66(185.90)	532.27(40.52)	524.65(61.47)
Day 1	85.43(38.43)	87.905(43.59)	97.03(17.98)	95.64(16.11)
Day 2	103.36 (42.13)	97.14(40.90)	114.58(10.32)	106(18.12)
Day 3	110.05(45.21)	103.61(44.66)	113.46(17.94)	109.23(15.83)
Day 4	127.08(44.15)	103.29(42.85)	126.66(13.34)	123.43(19.16)
Day 5.	90.29(40.61)	71.73(40.19)	80.55(18.09)	89.9(14.75)

Now we have found that the treatments itself does not lead to statistically significant outcomes, we will look in more detail to the data using multi-level regression analysis. Table 3 shows the average amount of points earned per person per day in the four treatments. They have the same pattern (increased performance until Thursday (Day 4), and drop on Friday (Day 5). The points earned do not differ significant (based on Mann-Whitney tests using p-value = 0.1) except for day 4 when treatment 5-NLB is significantly lower than the other treatments. However, groups of 5 without social information seem to peak on Wednesday. The experiments are performed during different semesters and each semester we find the same pattern. The drop on Friday might be caused by different priorities of the student participants at a large state university.

**Fig 4:**
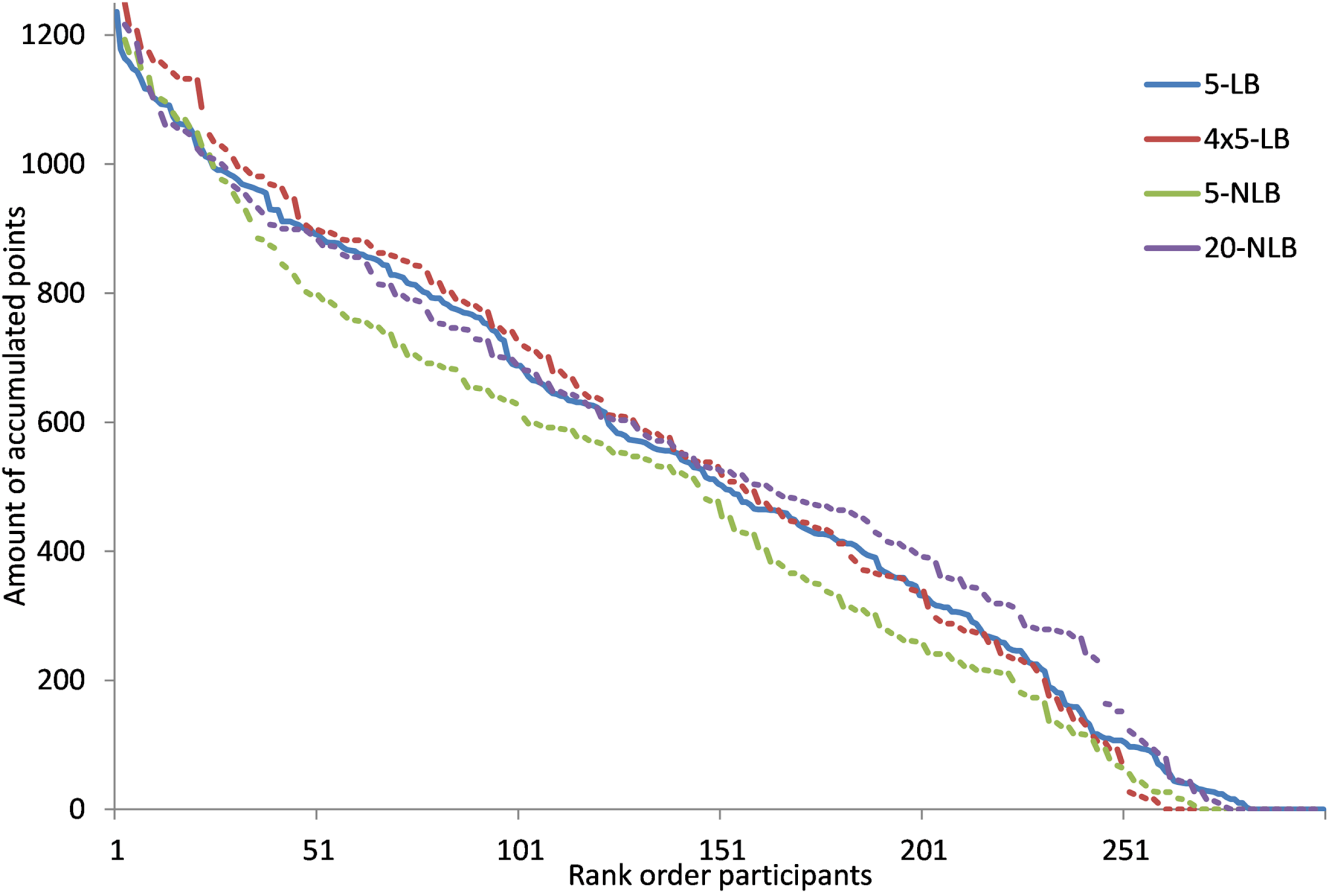
Distribution of points per person ordered by rank for the four different treatments. Participants could post messages and they made use of this option. A total of 346 messages were posted. The number of messages per day declined over the week (Fig 5). The content of the messages show that participants ask and answer questions on the workings of the experiments, lament about participants who are not participating, and in leaderboard conditions mention how they do compare to other groups. Some groups also mention the strategy to set reminders on their electronic devices when to login the experiments when points are available.

Fig 4 shows the distribution of points among the individuals in the four different treatments. The points will lay between 0 and 1250 points, and we rank the students from the highest to the lowest number of points they earned over 5 days. Since three treatments have 200 participants and one treatment 300 participants, we scaled the observations for the 200 participants to compare it with the treatment (5-LB) of 300 participants. Fig 4 demonstrates clearly that the distributions are very similar among the treatments. About 10 percent of the participants do not receive any noticeable number of points, while in each treatment there is also about 10% of them who earn 1000 points of more. Note that all participants opted in to an online experiment that would have a duration of 5 days.

**Fig 5:**
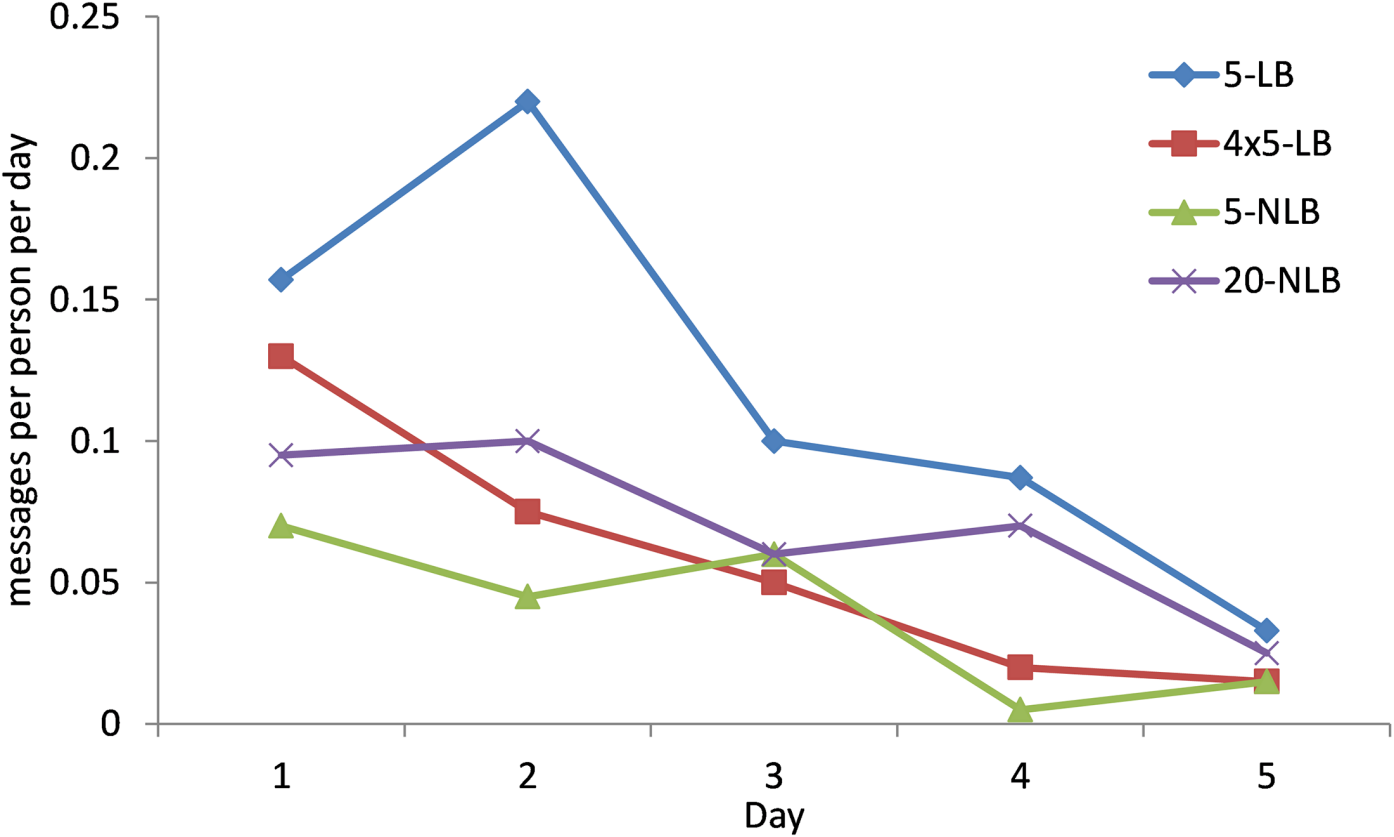
Average number of messages posted per person per day for each of the four treatments.

**Fig 6:**
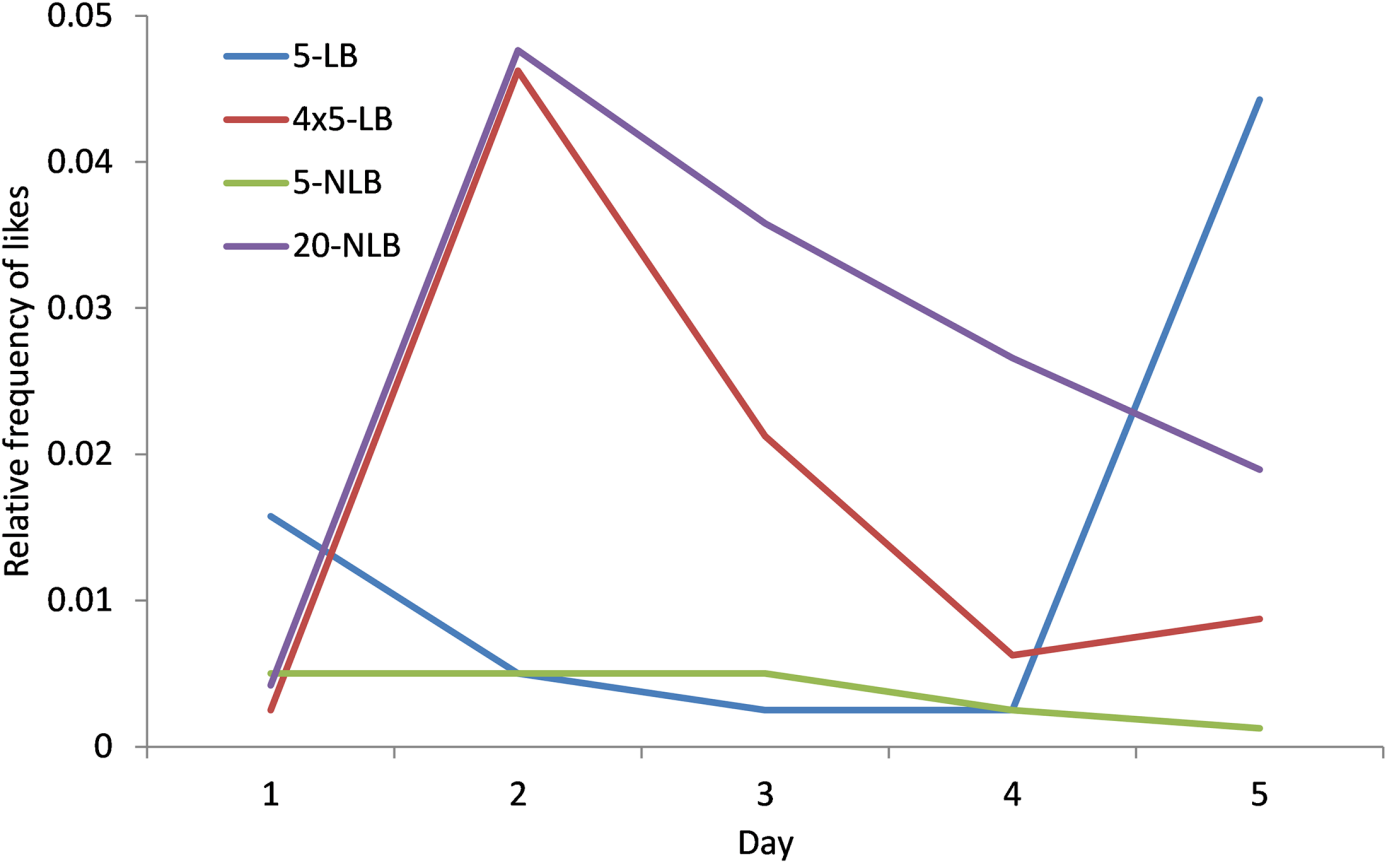
Mean likes for each day. Mean number of likes per person per day divided by the number of other persons in the (sub) group.

**Fig 7:**
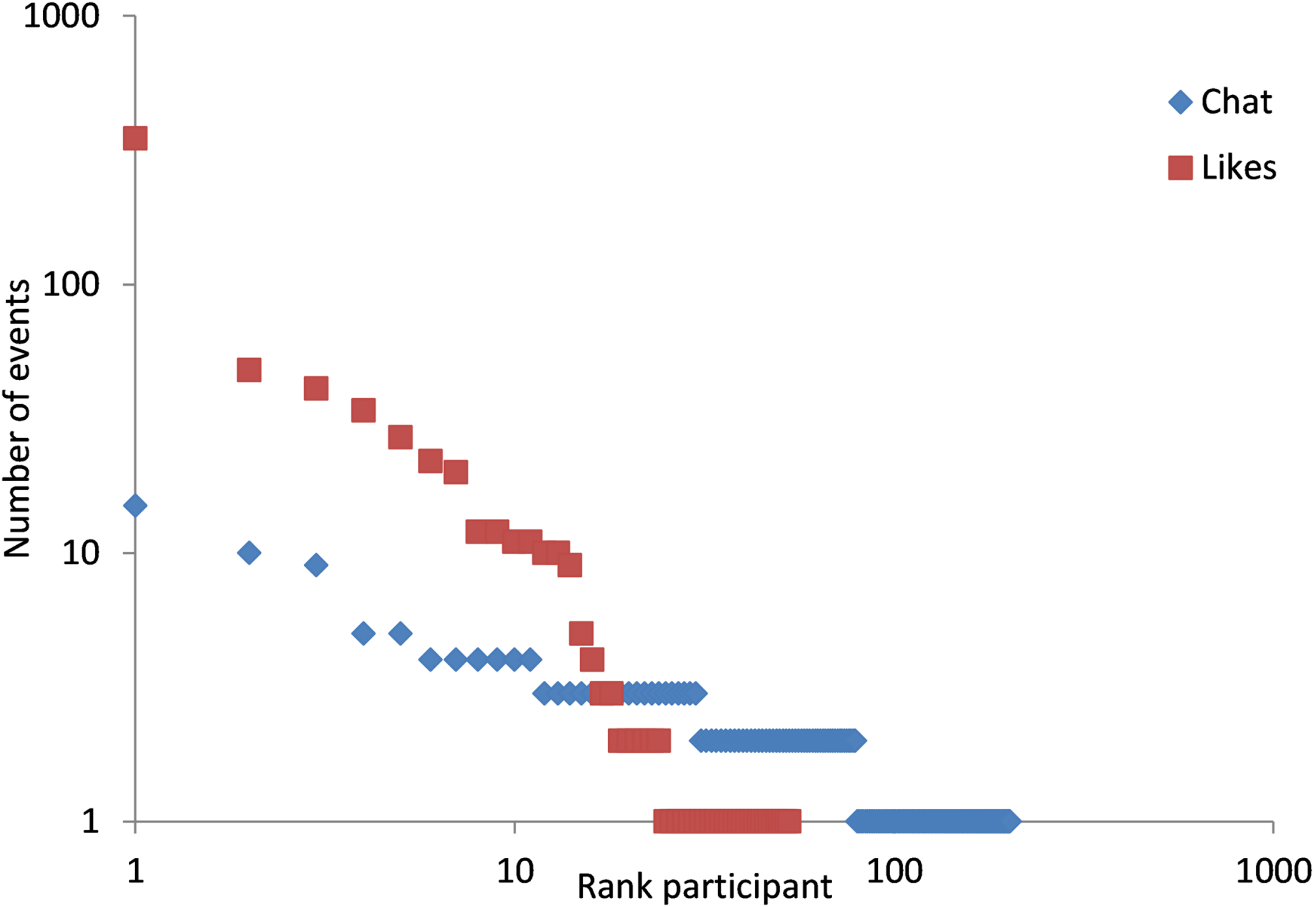
Distribution of likes. Log-Log plot of the number of likes per person for the 900 participants who are ranked in other of number of likes given.

There are also 673 likes given during the experiments. In groups of 20 participants give more likes per person, since they have more other participants to like their actions. Fig 6 reports the number of likes posted and scales the number of likes per person divided by the number of other participants in the group (19 for treatment 20-NLB, and 4 for treatments 5-LB, 4x5-LB and 5-NLB). We see that in all treatments, except treatment 5-NLB, there are days with many likes. Fig 7 shows that the distribution of Likes given is much more unequal compared to the posting of messages. The maximum number of messages is 15, while the maximum number of Likes given is 350. 202 participants posted a message while only 53 persons gave a Like to somebody.

We tested potential effects that explain the behavior of individuals during the experiments. In Fig 4 there was no significant difference between treatments at the individual and group level. But what is the effect of the communication and the posting of Likes? The nightly emails that participants received included the individual’s score, the group’s average score and the number of chat messages in the group. We performed a multi-level mixed-effects linear regression model using the individual level data (Table 4). In the first model (Model 1) we only include treatment dummies and the day of the week. We do not find significant effects of the independent variables. In the second model (Model 2), we do not include Day 5 (Friday) and now we find a positive effect of time, but no treatment effects. Model 3 includes Day 5 (Friday), but not Day 1 (Monday) since we include information participants in their nightly email. We include the number of points the individual earned the day before, as well as the average contribution of others in the group, the number of chat messages and the number of likes the others posted. We find that the total number of points earned during the previous day is a strong predictor for the amount of points for the current day. The points earned on average by others in the previous day have a negative impact, while the number of chat messages has a positive effect. In Model 4 we include a dummy variable for Day 5, the Friday, since we observe a sharp reduction in performance which might be caused by events outside the experiment (being it a Friday at a college campus). We also include dummies on whether groups that use leaderboards are ranked at the top 25% or the bottom 25%. We find now a positive effect of the actions of others in the previous day. This means that if others scored more points during the previous day, the participant increase the score in the current day. Note again that the participants get nightly emails with the performance of the group, which might stimulate people to increase their participation. We do not see an effect of chat messages or likes, treatment, or whether groups were ranked high or low. Finally, instead of individual treatments we control for the size of the group that shares the public good (20 for 20-NLB and 4x5-LB) and a dummy indicating there was a leaderboard (= 1) or not (= 0). Now we find a positive significant effect of the leaderboard. The leaderboard is predicted to increase the performance with 5 points per person per day, an increase of around 5%.

**Table 4:** A multilevel mixed-effects linear regression. Regression performed with the number of points that individuals collected during each day. We distinguish five models as discussed in the main text. The independent variables are the Points participants collected the previous day, group level information of the previous day (the number of Points per person, the number of chat messages, number of Likes), and dummies for the treatment participants were in. We controlled for group effects for performing a multi-level analysis where we indicated the groups participants in. The χ^2^ was not significant which means that there was no significant group effect on the error terms. For each variable of the regression we provide the estimated value, the standard deviation (between brackets), and the 95% confidence interval.

Independent variables	Model 1	Model 2	Model 3	Model 4	Model 5
Constant	104.345 (9.226)***[86.262; 122.428]	85.123 (9.469) *** [66.564; 103.682]	73.756 (8.269)*** [57.549; 89.962]	45.958(5.115)*** [35.933;55.983]	37.377(4.626)*** [28.311;46.444]
Day	0.195 (0.901)[-1.571;1.960]	9.425 (1.271)*** [6.934; 11.917]			
Points previous day			0.567 (0.014)*** [0.538; 0.595]	0.567 (0.015)*** [0.538; 0.595]	0.567 (0.014)*** [0.539; 0.595]
Points group previous day			-.303(0.046)***[-0.392; 0.214]	0.094(0.041)** [0.014; 0.175]	0.095(0.038)** [0.021; 0.169]
Chat messages group previous day			3.472(1.072)*** [1.371; 5.573]	0.346 (0.929) [-1.475; 2.168]	0.350 (0.929) [-1.470; 2.170]
Likes group previous day			0.033 (0.130) [-0.221; 0.287]	-0.0058 (0.077) [-0.157; 0.145]	-0.013 (0.075) [-0.160; 0.135]
Treatment 5-LB	-1.686 (9.718)[-20.733;17.361]	-2.205 (9.867) [-21.545; 17.134]	3.383 (7.468) [-11.255;18.021]	2.010 (3.271) [-4.400; 8.421]	
Treatment 5-NLB	-12.198(10.137) [-32.065; 7.669]	-10.703(10.309) [-30.908; 9.501]	-6.449 (7.888) [-21.909;9.011]	-6.822 (3.993)* -6.822048 [-14.649;1.005]	
Treatment 20-NLB	1.487 (12.475) [-22.964; 25.938]	4.195(12.6143) [-20.529; 28.919]	-1.547 (9.567) [-20.298; 17.205]	-2.566 (3.957) [-10.321;5.189]	
Day 5 dummy				-40.209 (2.874)*** [-45.843; -34.576]	-40.229 (2.854)*** [-45.823; -34.634]
Top rank previous day				-2.207 (4.171) [-10.382; 5.969]	
Low rank previous day				-1.681 (4.031) [-9.582; 6.220]	
Leaderboard					7.964(3.250)** [1.593; 14.334]
Size group sharing public good					0.340 (0.217) [-0.085; 0.764]
Number of observations	4500	3600	3580	3580	3580
Log likelihood	-26486.679	-21189.035	-20398.147	-20306.632	-20306.921
Wald χ^2^	3.62(p = 0.460)	57.88(p<0.01)	1586.94(p<0.01)	2052.68(p<0.01)	2051.77(p<0.01)
χ^2^	138.72(p<0.01)	100.02(p<0.01)	10.72(p<0.01)	0 (p = 1.0)	0(p = 1.0)

The *, ** or *** next to the standard deviation means a p-value smaller than 0.1, 0.05 or 0.01, respectively.

In sum, we still do not find specific treatment effects if we control for the days of the weak, and the information participants get. However, the use of leaderboard itself leads to a small increase (5%) of performance. We do find that a more participation by others in the previous day stimulate the actions of the participants, which may indicate conditional cooperation. This means that participants cooperate if others do too.

## Discussion

This paper presented the first results of a new experimental environment where participants invest time in the public good during a period of days. We find a major inequality in the amount of participation among the participants, even though they signed up for the experiment just days before and received a reminder digest email every evening. When participants have to decide to invest their time to contribute to the public good, this investment of time faces competition with alternative activities. This is not the case when subjects participate in an experiment in the laboratory. Using other online platforms like Amazon’s Mechanical Turk to conduct group experiments for a brief period of time (e.g., one hour) also introduces a limited amount of competition for alternative activities as shown by the fact that 10% of participants also drop out during the experiment [24].

Adding a leaderboard to the experiment had a small positive effect if we control of group attributes such as the number of chat messages and likes as well as group size. Thus we can replicate the observed effect of [23] that intergroup competition–without monetary incentives to win, increases the level of cooperation. We do find an effect of the amount of points earned on average by other group members in the previous day on the actions of individuals in the current day. This result confirms the finding in many other public good experiments that many participants are conditional cooperative [26]. Conditional cooperation means that individuals cooperate if they expect others will cooperate. Thus participants are influenced by information about participation in their own group, and by the relative performance of their group compared to other groups.

The effect of the individual treatments is not significant. This might be caused by a limitation of our experiment, which namely that our experiments participants are not known to each other while recent studies find the important of the influence of the strength of peers [27]. Nevertheless, the conditional cooperation effect is in line with other online experiments. Some studies such as [15] and [16] find statistical significant effects among hundred thousands or millions of participants where the absolute effect is very small. In those cases information about voting or energy use of their peers affect the decisions of individuals but the group size of such public group size is technically hundreds of millions (voting affect a nation, and energy use affect global climate change). In our controlled web-based experiment we could test more variations and found no significant effects among the individual treatments. A possible reason for the lack of significant effects in our experiment is the lack of social context experienced by the participants (they interact with fellow students, but not their own social network and not their own group identity) [27]. Only by combining treatments and control for group attributes, we could replicate the small effect from laboratory experiments by [23]. A hypothesis is that the social context is critical for collaboration online over a number of days, where people have to come back to check updates. This suggests that it is important for the effectiveness of social influence that the information is socially embedded.

To conclude, we find that actions of other group members have a positive effect, and we do find a positive effect of information on relative group performance. For future work when we include physical actions we expect that it is vital to grow social groups from existing social networks, instead of assigning participants into particular roles. It is important to facilitate social network traffic that leaves information about the activity of others and relative to other groups. One is willing to contribute when there is evidence that others are also contributing.

## Supporting Information

S1 File Data from the Experiment. (XLSX)

## References

[1] Ostrom E. Governing the Commons: The Evolution of Institutions for Collective Action. New York: Cambridge University Press; 1990.

[2] Poteete AM, Janssen MA, Ostrom E. Working Together: Collective Action, the Commons and Multiple Methods in Practice. Princeton, NJ: Princeton University Press; 2010.

[3] Gibson CC, Ostrom E, Ahn TK. The concept of scale and the human dimensions of global change: a survey. Ecological Economics 2000; 32(2), 217–239.

[4] Ostrom E. Polycentric Systems for Coping with Collective Action and Global Environmental Change. Global Environmental Change 2010; 20(4), 550–557.

[5] Froehlich J, Larson E, Gupta S, Cohn G, Reynolds M, Patel SN. Disaggregated End-Use Energy Sensing for the Smart Grid. IEEE Pervasive Computing 2011; 10(1): 28–39.

[6] Larson E, Froehlich J, Campbell T, Haggerty C, Fogarty J, Patel SN. Disaggregated Water Sensing from a Single, Pressure-based Sensor: An Extended Analysis of HydroSense using Staged Experiments. Pervasive and Mobile Computing 2012; 8(1): 82–102.

[7] Mattern F, Staake T, Weiss M. ICT for Green–How Computers Can Help Us to Conserve Energy. E-Energy’10 Proceedings of the 1st International Conference on Energy-Efficient Computing and Networking, 1–10, ACM New York, NY, USA, 2010.

[8] Hauber-Davidson G, Idris E. Smart Water Metering. Water 2006; 33(3): 38–41.

[9] Froehlich J, Dillahunt T, Mankoff J, Consolvo S, Harrison B, Landay JA. UbiGreen: investigating a mobile tool for tracking and supporting green transportation habits. CHI’ 09 Proceedings of the 27th international conference on Human factors in computing systems 2009; 1043–1052.

[10] Hay GJ, Kyle C, Hemachandran B, Chen G, Rahman MM, Fung TS, et al Geospatial Technologies to Improve Urban Energy Efficiency. Remote Sensing 2011; 3: 1380–1405.

[11] Marlow C, Byron L, Lento T, Rosenn I. Maintained relationships on facebook. http://overstated.net/2009/03/09/maintained-relationships-on-facebook, 2009 Accessed January 20, 2016.

[12] Dickinson JL, Crain RL, Reeve HK, Schuld JP. Can evolutionary design of social networks make it easier to be ‘green’? Trends in Ecology & Evolution 2013; 28(9): 561–569. 23787089 10.1016/j.tree.2013.05.011

[13] Ballew MT, Omoto AM, Winter PL. Using Web 2.0 and Social Media Technologies to Foster Proenvironmental Action, Sustainability 2015; 7(8): 10620–10648.

[14] Mankoff J, Fussell SR, Dillahunt T, Glaves R, Grevet C, Johnson M, et al. StepGreen.org: Increasing Energy Saving Behaviors via Social Networks. Proceedings of the Fourth International AAAI Conference on Weblogs and Social Media, 2010; 106–113.

[15] Allcott H. Social norms and energy conservation. Journal of Public Economics 2011; 95(9–10): 1082–1095.

[16] Bond RM, Fariss CJ, Jones JJ, Kramer ADI, Marlow C, Settle JE, et al A 61-million-person experiment in social influence and political mobilization, Nature 2012; 489, 295–298. 10.1038/nature11421 22972300 PMC3834737

[17] Chaudhuri A. Sustaining cooperation in laboratory public good experiments: a selective survey of the literature. Experimental Economics 2011; 14(1): 47–83.

[18] Neugebauer T, Perote J, Schmidt U, Loos M. Selfish-boas conditional cooperation: On the decline of contributions in repeated public goods experiments. Journal of Economic Psychology 2009; 30(1): 52–60.

[19] Paolacci G, Chandler J, Ipeirotis PG. Running experiments on Amazon Mechanical Turk. Judgment and Decision Making 2010; 5(5): 411–419

[20] Rand DG, Arbesman S, Christakis NA. Dynamic social networks promote cooperation in experiments with humans. Proceedings of the National Academy of Sciences USA 2011; 108(48): 19193–19198. 10.1073/pnas.1108243108 PMC3228461 22084103

[21] Mason W, Suri S. Conducting behavioral research on Amazon’s Mechanical Turk. Behavior Research Methods 2012; 44(1): 1–23. 10.3758/s13428-011-0124-6 21717266

[22] Wu L, Baggio JA, Janssen MA. The Role of Diverse Strategies in Sustainable Knowledge Production. PLoS ONE 2016; 11(3): e0149151 10.1371/journal.pone.0149151 26934733 PMC4775042

[23] Tan JHW, Bolle F. Team competition and the public goods game. Economics Letters 2007; 96: 133–139.

[24] Suri S, Watts DJ, Cooperation and Contagion in Web-Based, Networked Public Goods Experiments. PLoS ONE 2011; 6(3): e16836 10.1371/journal.pone.0016836 21412431 PMC3055889

[25] Olson M. The Logic of Collective Action: Public Goods and the Theory of Groups, Harvard University Press: Cambridge, MA; 1965.

[26] Fischbacher U, Gächter S, Fehr E. Are people conditionally cooperative? Evidence from a public goods experiment. Economics Letters 2001; 71(3), 397–404.

[27] Aral S, Walker D. Tie Strength, Embeddedness, and Social Influence: A Large-Scale Networked Experiment, Management Science 2014; 60(6): 1352–1370.

